# Cross-sectional and prospective relationship between physical activity and depression symptoms

**DOI:** 10.1038/s41598-020-72987-4

**Published:** 2020-09-30

**Authors:** Adilson Marques, Joana Bordado, Miguel Peralta, Elvio R. Gouveia, Riki Tesler, Yolanda Demetriou, Diego Gomez Baya

**Affiliations:** 1 CIPER, Faculdade de Motricidade Humana, Univerisdade de Lisboa, Estrada da Costa, Cruz Quebrada, 1499-002 Lisboa, Portugal; 2 ISAMB, Faculdade de Medicina, Univerisdade de Lisboa, Lisboa, Portugal; 3 University of Huelva, Huelva, Spain; 4 Faculdade de Motricidade Humana, Univerisidade de Lisboa, Lisboa, Portugal; 5 Interactive Technologies Institute, LARSyS, Funchal, Portugal; 6 Department of Health Systems Management, Faculty of Health Sciences, Ariel University, Ariel, Israel; 7 Faculty of Sport and Health Sciences, Technical University of Munich, Munich, Germany

**Keywords:** Psychology, Risk factors

## Abstract

This study aimed to analyse the cross-sectional and prospective relationship between moderate and vigorous physical activity (PA) and depression symptoms. This study analysed 32,392 European late middle-aged to older adults, from 14 European countries across a 4-year follow-up. Data was collected in the fourth (in 2011) and sixth (in 2015) wave, from the Survey of Health, Ageing and Retirement in Europe (SHARE). For the present analysis, participants were considered who responded to the EURO-D 12-item scale of depression symptoms and reported the intensity and frequency of PA. ANCOVAs were conducted to assess the cross-sectional and prospective associations. For both men and women, engaging in moderate or vigorous PA in 2011 was associated with a lower score of depression in 2011 and 2015. From the prospective analysis, moderate and vigorous PA in 2011 was inversely associated with the score of depression. This association remains significant in the fully adjusted for self-rated health, sociodemographic characteristics, and the presence of chronic diseases. Moderate and vigorous PA at least once a week is negatively related to the score of depression, both in men and women. PA is negatively associated with depression symptoms, and from prospective analysis PA predicts lower depression scores 4 years later.

## Introduction

The prevalence of depression has been rising globally with an estimate that more than 300 million people suffer from this condition^1^. Additionally, it is estimated that about one-third of depressive cases evolve into moderate-to-severe symptomatic depressions^2^. Depression, following heart disease, is projected to be the second-largest contributor to the global burden of disease by 2020^3^, and it is already considered as the greatest non-fatal disease contributory to the loss of health^1^.

The prevalence of depression is the highest among the 55–74 years old age group^1^. Comparing with other chronic diseases, it appears that older people suffering from depression are less functional^4^. However, as other age-related diseases might have concomitant symptoms, depression appears to be often neglected and even underdiagnosed in primary health care^5^. In late middle aged to older people, serious health consequences are associated with untreated or poorly treated depressions, including increased mortality^6^.

The development of depression seems to increase with low levels of physical activity (PA)^7^. Strong evidence exists regarding the positive effects of PA for both the prevention and treatment of depression, being even considered an alternative to antidepressant medication^8^. Depression and PA have been the subject of several studies, however, often the sample size is small and restricted to one country or specific area, and most studies do not control for moderate and vigorous PA intensity levels, respectively. Thus, the present study aims to analyse the cross-sectional and prospective relationship between different PA intensity levels and depression symptoms in a large sample of European late middle aged to older adults.

## Methods

This study analysed data from the Survey of Health, Ageing and Retirement in Europe (SHARE) from wave 4 (performed in 2011) and wave 6 (performed in 2015). SHARE is a multidisciplinary and cross-national survey resulting from the need to study and provide answers regarding the growing and upcoming challenges of population ageing. SHARE’s data collection has been conducted biannually, since 2000. Including about 140,000 individuals aged 50 or older, from European countries and Israel, SHARE is a multicultural database of health microdata, socio-economic status, social and family networks. In the present study, including waves 4 and 6, participants were from the 14 following countries: Austria, Germany, Sweden, Spain, Italy, France, Denmark, Switzerland, Belgium, Czech Republic, Poland, Portugal, Slovenia, and Estonia^9^. The total sample consisted of 34,772 men and women that participated in both wave 4 and wave 6. For the present study, the final sample comprised 32,392 participants (13,511 men, 18,881 women) that responded to the 12-item depression scale and reported on PA.

All interviews were conducted by trained interviewers at the participant’s house location and had a duration of approximately 90 min. SHARE’s questions encompass a large spectrum of subjects, such as health, and social variables. For the research community, SHARE’s data is available free of charge (www.share-project.org)^9^. Participants were informed about the objectives of the study and informed written consent was obtained. The study was in accordance with the ethical standards in sport and exercise science research^10^. All methods were performed in accordance with the relevant guidelines and regulations; and participation was anonymous and there were no incentives for participation. The SHARE’s protocol was approved by the Ethics Committee of the University of Mannheim and by the Ethics Council of the Max-Planck-Society for the Advancement of Science.

### Measurements

#### Covariates

Individual’s characteristics were measured at the baseline and include age, sex, marital status, education level, living place, country, self-rated health and the number of chronic diseases. Age was recoded into 50–64 years, 65–79 years, and ≥ 80 years. According to the International Standard Classification of Education-97 (ISCED- 97) codes, education was collected and categorized in three groups: (1) low education level, including participants with no education or ISCED-97 codes 1 and 2; (2) middle educational level, which included participants with ISCED-97 codes 3 and 4; (3) high education level, which included participants with ISCED-97 codes 5 and 6. Marital status was coded into married, including a registered partnership or living together; and not married, including single, separated, divorced or widowed. To determine the participants’ living place, they were asked to report on whether they lived in a big city, a suburb or the outskirts of a big city, a large town, a small town, or in rural area or village. Self-related health was accessed through a single-item question about their general health perception, using a 5-point scale response (excellent, very good, good, fair and poor). This scale was inverted for data analysis; therefore, higher values represent better health perception.

#### Number of chronic diseases

To determine the number of chronic medical diseases participants were asked to report the presence or absence of diseases that were previously diagnosed by a doctor, based on a list with 14 listed diseases (heartattack, hypertension, cholesterol, stroke, diabetes, lung disease, asthma, arthritis, osteoporosis, cancer, ulcer, Parkinson disease, cataracts, hip femoral fracture). Participants could also add other medical conditions, diagnosed by a doctor, unnamed in the list^11^. In order to produce a single score, the number of chronic diseases was summed as performed previously^12,13^.

#### Physical activity

With the purpose to measure intensity and frequency of PA, participants were asked to report on how many days per week they practiced moderate PA such as, brisk walking, gardening or household activities; as well as the frequency of vigorous PA, including activities like hiking, sports, carrying heavy loads. To measure its frequency, for both moderate and vigorous activities, the responses were categorized into: (1) more than once a week, (2) once a week, (3) up to three times a month, and (4) hardly ever or never. The responses up to three times a month and hardly ever or never were grouped into a category named “less than once a week” as performed previously^12,13^.

#### Depression

The EURO-D 12-item scale was developed and validated to assess and compare cross-nationally symptoms of depression in Europe^14^. This scale asks about the presence or absence of depression symptoms in 12 domains s that are: depressed mood, pessimism, suicidality, guilt, sleep, interest, irritability, appetite, fatigue, concentration, enjoyment, and tearfulness. Each depression’s domain is scored one if present and zero if absent. The sum of all domains results in the total score between 0 and 12. In each of the EURODEP study sites the score’s validation was carried out and an optimal cut-off point of 4 or more points to diagnose a clinically significant depression was demonstrated^11,14^.

### Data analysis

Descriptive statistics were calculated (means, standard deviation, and percentages) for the entire sample, and were stratified by sex. ANOVA tests were performed, to analyse the bivariate cross-sectional and prospective relationships between, moderate and vigorous PA and the score of depression symptoms, in 2011 and 2015. Using ANCOVA, the cross-sectional and prospective association between moderate and vigorous PA, with the score of depression was analysed. For the cross-sectional analysis, different models were performed. Model 1 is the unadjusted analysis between the PA and the depression score. Model 2 was adjusted for age, marital status, educational level, living place, country, and self-rated health. Model 3 was further adjusted for the number of chronic diseases. A fourth model was created for prospective analysis. Model 4 was further adjusted from Model 3’s variables, depression score at baseline. Women are more prone to have depression symptoms than men^15^, therefore, analysis were stratified by sex. Binary logistic regressions were conducted to assess the association between PA trajectory over the years and depression symptoms. An interaction effect was observed between age (50–64 years, 65–80 years, and ≥ 80 years) and PA, on depression symptoms. Thus, analyses were stratified by sex, age-groups. Data analysis was performed using SPSS 25. Statistical significance was set at two-sided p < 0.05.

## Results

Table 1 shows the sociodemographic characteristics of the participants. The depression score was significantly higher in women than in men, both in 2011 (*p* < 0.001) and in 2015 (*p* < 0.011). Moreover, significantly more women than men (33.8% vs. 18.4% in 2011; 34% vs. 19.6% in 2015) presented depressive symptoms (≥ 4 EURO depression score). Regarding the practice of PA, men engaged significantly more often in moderate PA (*p* < 0.001) and vigorous PA (*p* < 0.001) per week than women.

**Table 1:** Participants’ characteristics (n = 32,392).

	2011 (% or M ± SD)			2015 (% or M ± SD)		
	Men	Women	*p*	Men	Women	*p*
Age	65.4 ± 9.0	65.0 ± 9.7	< 0.001	69.4 ± 9.3	68.9 ± 10.0	< 0.001
**Age group**			< 0.001			< 0.001
50–64 years	49.5	51.6		33.3	36.2	
65–79 years	43.0	39.9		51.3	47.9	
≥ 80 years	7.6	8.5		15.4	15.9	
**Education**			< 0.001			< 0.001
Low	36.3	42.3		36.3	42.3	
Middle	40.4	38.2		40.4	38.1	
High	23.3	19.5		23.4	19.5	
**Marital status**			< 0.001			< 0.001
Not married	22.9	39.1		42.4	67.8	
Married	77.1	60.9		57.6	32.2	
**Living place**			< 0.001			< 0.001
Big city	13.1	15.2		13.5	15.2	
Suburbs of a big city	10.1	9.5		11.4	9.8	
Large town	14.8	16.7		13.7	15.1	
Small town	24.4	24.4		25.1	25.0	
Rural area	37.6	34.2		36.3	35.0	
EURO depression scale (score)	1.9 ± 1.9	2.9 ± 2.3	< 0.001	2.0 ± 2.0	2.9 ± 2.3	< 0.001
Depressive symptoms (score ≥ 4)	18.4	33.8	< 0.001	19.6	34.0	< 0.001
Self-rated health	2.9 ± 1.1	2.8 ± 1.1	< 0.001	2.8 ± 1.0	2.7 ± 1.0	< 0.001
**Number of chronic diseases**	1.6 ± 1.4	1.7 ± 1.5	< 0.001	1.7 ± 1.5	2.0 ± 1.7	< 0.001
Moderate PALess than once a week	14.1	17.1	< 0.001	16.1	18.7	< 0.001
Once a week	13.6	14.2		11.9	12.6	
More than once a week	72.3	68.7		72.0	68.7	
**Vigorous PA**			< 0.001			< 0.001
Less than once a week	45.1	55.6		49.8	58.0	
Once a week	14.4	14.4		13.5	13.1	
More than once a week	40.5	30.0		36.7	28.9	

*M* media, *SD* standard deviation, *PA* physical activity.

^a^Tested by Chi Square.

^b^Tested by t test.

Table 2 presents the cross-sectional parameters estimates of depression score according to PA intensity and frequency. For both genders, moderate and vigorous PA were negatively associated with the depression score.In the fully adjusted model (analyses adjusted for age, marital status, educational level, living place, country, self-rated health, and the number of chronic diseases), for men, practicing moderate PA once a week (β = − 0.37, 95% CI − 0.50, − 0.24, p < 0.001) and more than once a week (β = − 0.56, 95% CI − 0.66, − 0.46, p < 0.001) was negatively associated with the score of depression. For vigorous PA, similar results were observed (once a week: β = − 0.29, 95% CI − 0.40, − 0.17, p < 0.001; more than once a week: β = − 0.32, 95% CI − 0.41, − 0.24, p < 0.001), PA was negatively associated with depression score. Regarding women, practicing moderate PA once a week (β = − 0.37, 95% CI − 0.50, − 0.25, p < 0.001) and more than once a week (β = − 0.56, 95% CI − 0.65, − 0.47, p < 0.001) was also negatively associated with a lower score of depression. Similarly, this results were also observed for practicing vigorous PA (once a week: β = − 0.35, 95% CI − 0.46, − 0.24, p < 0.001; more than once a week: β = − 0.17, 95% CI − 0.25, − 0.08, p < 0.001).

**Table 2:** Cross-sectional parameters estimate of depression score according to physical activity intensity levels and frequency.

	Parameters estimates of predicting the score of depression in 2011		
	Model 1 β (95% CI)	Model 2 β (95% CI)	Model 3 β (95% CI)
**Men**			
**MPA in 2011**			
Less than once a week	ref	ref	Ref
Once a week	− 0.97 (− 1.06, − 0.87)*	− 0.37 (− 0.51, − 0.24)*	− 0.37 (− 0.50, − 0.24)*
More than once a week	− 1.31 (− 1.38, − 1.24)*	− 0.56 (− 0.66, − 0.46)*	− 0.56 (− 0.66, − 0.46)*
**VPA in 2011**			
Less than once a week	ref	ref	Ref
Once a week	− 0.75 (− 0.83, − 0.67)*	− 0.31 (− 0.43, − 0.20)*	− 0.29 (− 0.40, − 0.17)*
More than once a week	− 0.87 (− 0.92, − 0.81)*	− 0.36 (− 0.45, − 0.28)*	− 0.32 (− 0.41, − 0.24)*
**Women**			
**MPA in 2011**			
Less than once a week	ref	ref	Ref
Once a week	− 1.04 (− 1.13, − 0.95)*	− 0.41 (− 0.53, − 0.28)*	− 0.37 (− 0.50, − 0.25)*
More than once a week	− 1.41 (− 1.47, − 1.34)*	− 0.58 (− 0.67, − 0.49)*	− 0.56 (− 0.65, − 0.47)*
**VPA in 2011**			
Less than once a week	ref	ref	Ref
Once a week	− 0.89 (− 0.97, − 0.82)*	− 0.42 (− 0.53, − 0.31)*	− 0.35 (− 0.46, − 0.24)*
More than once a week	− 0.88 (− 0.93, − 0.81)*	− 0.21 (− 0.29, − 0.12)*	− 0.17 (− 0.25, − 0.08)*

Model 1: Unadjusted analyses.

Model 2: Analyses were adjusted for age, marital status, educational level, living place, country and self-rated health.

Model 3: Analyses were adjusted for age, marital status, educational level, living place, country, self-rated health, and the number of chronic diseases.

*MPA* moderate physical activity, *VPA* vigorous physical activity, *CI* confidence interval.

*p < 0.001.

Table 3 shows the prospective analysis of parameters estimates predicting the score of depression. Moderate and vigorous PA practice, once or more than once a week were negatively associated with the depression score 4 years later. Even when the model was fully adjusted (model 3), this relationship remained significant. Regarding men, engaging in moderate PA (more than once a week: β = − 0.26, 95% CI − 0.40, − 0.12, p < 0.01) and vigorous PA showed a significant reduction of depression score (more than once a week: β = − 0.13, 95% CI − 0.24, − 0.03, p < 0.05). Among women, moderate PA (once a week: β = − 0.26, 95% CI − 0.42, − 0.10, p < 0.01; more than once a week: β = − 0.52, 95% CI − 0.64, − 0.40, p < 0.01) and vigorous PA (more than once a week: β = − 0.20, 95% CI − 0.31, − 0.10, p < 0.001) significantly reduced the depression score. In general, prospective analysis showed that PA predicts lower depression scores 4 years later.

**Table 3:** Prospective parameters estimate of Depression Score to physical activity intensity levels and frequency.

	Parameters estimates of predicting the score of depression in 2015		
	Model 1 β (95% CI)	Model 2 β (95% CI)	Model 3 β (95% CI)
**Men**			
**MPA in 2011**			
Less than once a week	Ref	Ref	Ref
Once a week	− 0.64 (− 0.77, − 0.51)***	− 0.25 (− 0.44, − 0.06)**	− 0.12 (− 0.30, 0.06)
More than once a week	− 0.83 (− 0.93, − 0.74)***	− 0.43 (− 0.57, − 0.28)***	− 0.26 (− 0.40, − 0.12)**
**VPA in 2011**			
Less than once a week	Ref	Ref	Ref
Once a week	− 0.55 (− 0.65, − 0.45)***	− 0.15 (− 0.30, 0.01)	− 0.07 (− 0.22, 0.07)
More than once a week	− 0.63 (− 0.70, − 0.56)***	− 0.21 (− 0.32, − 0.09)***	− 0.13 (− 0.24, − 0.03)*
**Women**			
**MPA in 2011**			
Less than once a week	Ref	Ref	Ref
Once a week	− 0.82 (− 0.93, − 0.70)***	− 0.35 (− 0.52, − 0.18)***	− 0.26 (− 0.42, − 0.10)**
More than once a week	− 1.23 (− 1.32, − 1.15)***	− 0.70 (− 0.83, − 0.57)***	− 0.52 (− 0.64, − 0.40)**
**VPA in 2011**			
Less than once a week	Ref	Ref	Ref
Once a week	− 0.66 (− 0.76, − 0.57)***	− 0.26 (− 0.40, − 0.12)***	− 0.13 (− 0.26, − 0.01)
More than once a week	− 0.79 (− 0.86, − 0.71)***	− 0.31 (− 0.42, − 0.20)***	− 0.20 (− 0.31, − 0.10)***

Model 1: Unadjusted analyses.

Model 2: Analyses were adjusted for age, marital status, educational level, living place, country, self-rated health, and the number of chronic diseases.

Model 3: Analyses were adjusted for age, marital status, educational level, living place, country, self-rated health, the number of chronic diseases in 2011, and depression scores at baseline.

*MPA* moderate physical activity, *VPA* vigorous physical activity, *CI* confidence interval.

*p < 0.05**, *p* < 0.01, **p < 0.001.

**Figure 1:**
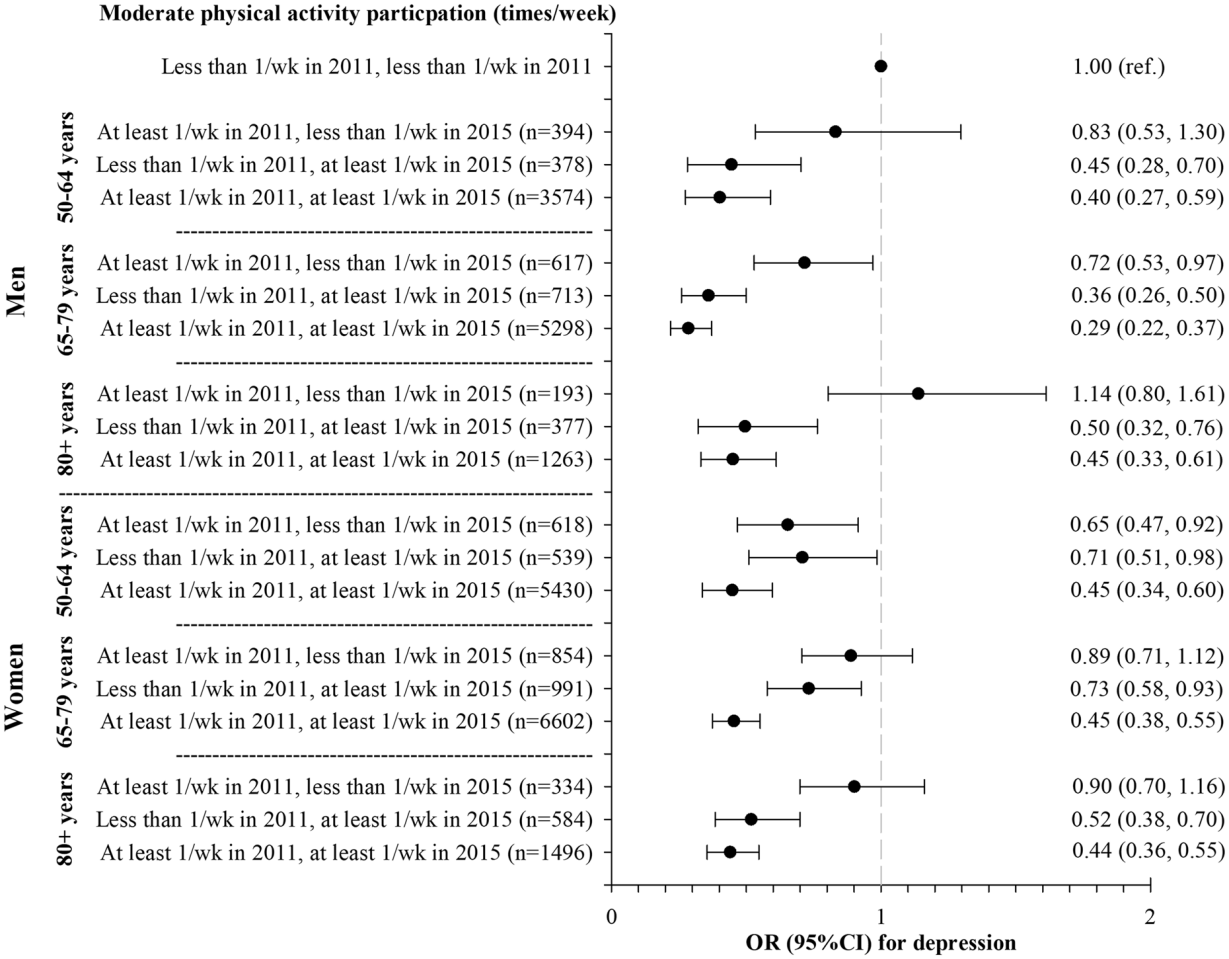
Relationship between moderate physical activity trajectory from 2011 to 2015 and depression symptoms (depression score ≥4). Analyses were adjusted for age, marital status, educational level, living place, country, self-rated health, the number of chronic diseases, and depression scores at baseline. *wk* week, *OR* odds ratio, *CI* confidence interval.

**Figure 2:**
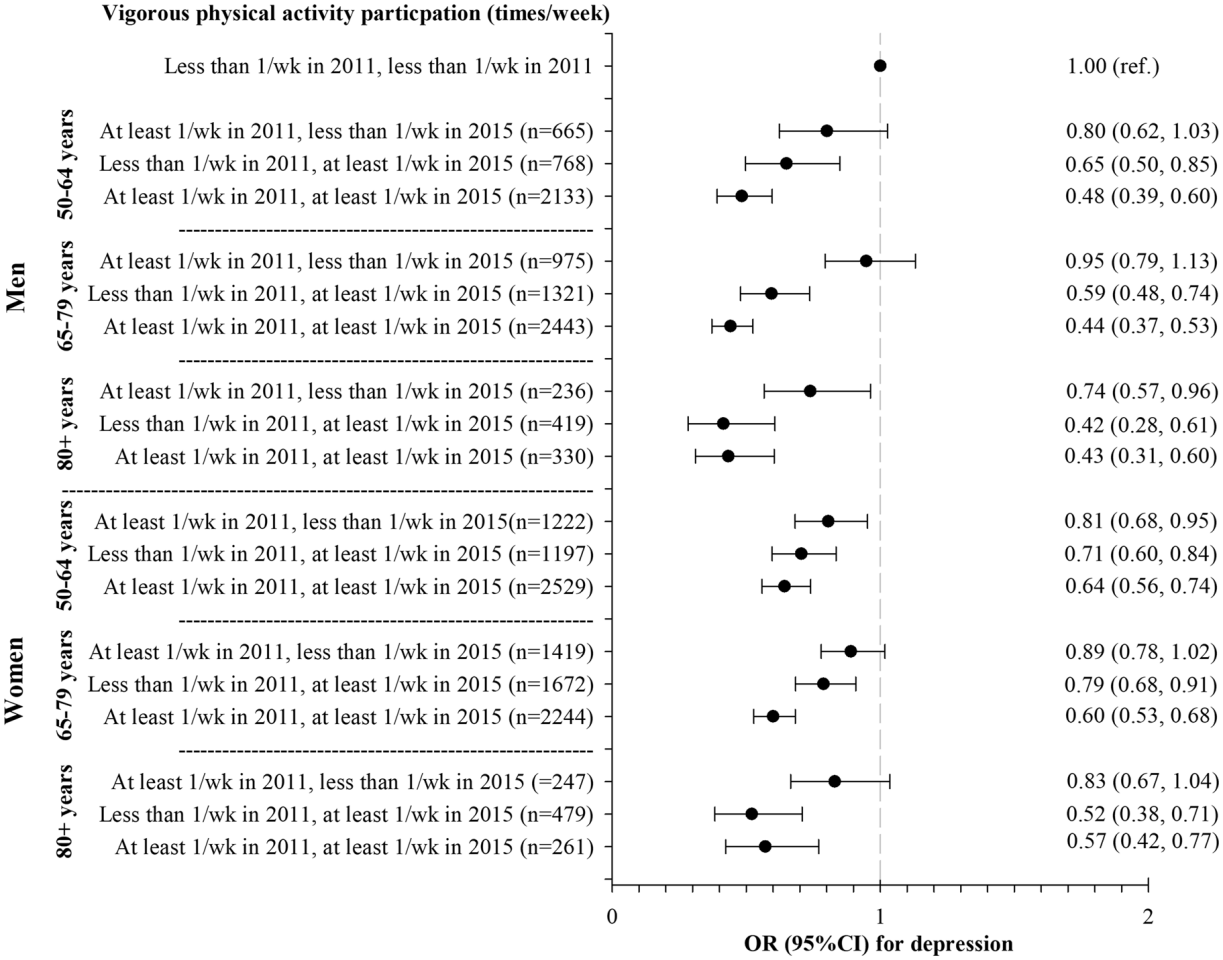
Relationship between vigorous physical activity trajectory from 2011 to 2015 and depression symptoms (depression score ≥4). Analyses were adjusted for age, marital status, educational level, living place, country, self-rated health, the number of chronic diseases, and depression scores at baseline. *wk* week, *OR* odds ratio, *CI* confidence interval.

Figures 1 and 2 present the results of the logistic regression analyses of the relationship between PA trajectory from 2011 to 2015 and depression symptoms (depression score ≥ 4). For moderate PA, in all age groups for both men and women, being active in the present decreases significantly the odds of having depressive symptoms, even if they hadn’t practiced PA less than once a week, compared to those consistently less active (engage in PA less than once a week). Except for women aged 50–64 years, those who practiced PA at least once a week but stopped practicing do not differ significantly from those who were consistently less active. Very similar results were observed for vigorous PA in all age groups for men and women. Those who engage in vigorous PA at least once a week consistently or only in the present had less likelihood of having depressive symptoms when compared to those consistently less active.

## Discussion

The cross-sectional and prospective relationship between PA and the depression score were analysed through a 4-year period (2011–2015) in a large sample of European late middle aged to older adults from 14 countries. The main result of this study emphasizes that those who practice PA less than once a week are more prone to have higher scores of depression, than those who engage in PA at least once a week. This result was found for both sexes in the cross-sectional and prospective analysis. Moreover, engage in moderate or vigorous PA at least once a week consistently or only in the present is related to lower odds of having depressive symptoms when compared to those consistently less active.

Emphasizing the importance of regular PA practice, these study results corroborate the current literature associating PA with lower depression scores or depression symptomatology^16,17^. The positive effects of PA in depression symptomology are well documented^16,18,19^, it is even considered by some authors as comparable to antidepressant treatments^20^. A review of four meta-analyses reached the conclusion that PA effects seem to be similar to those of medication and psychotherapy for mild-to-moderate depression^21^. This review, also noted that PA seems to be a valuable complement to standard clinical approaches for severe depression^21^. Furthermore, in late life treatment-resistant depressions it was found that combining PA with antidepressant medication seems to have a promising beneficial effect^22^. Nonetheless, more detailed clinical approaches are necessary for optimal PA and medication prescription to strongly support its clinical administration^23^.

In order to better understand the relationship between PA and depression, it is important to consider the characteristics of PA, such as frequency and intensity^24^. However, most studies only account for the amount of PA and intensity and frequency is rarely considered at the same time^19^. Acknowledging this gap, the present study provides PA frequency and intensity differentiation. Regarding intensity, it was found that moderate PA had a stronger inverse association with the depression score, than vigorous PA, in both cross-sectional and prospective analysis. This may be related to the proportion of older adults practicing these intensities of PA. Older adults are not expected to have the capacity to practice high amounts of vigorous PA, thus moderate PA is a more achievable way to practice PA and collect its benefits. Regarding frequency, practicing PA more than once a week had a stronger inverse association with the depression score, than practicing once a week, in both cross-sectional and prospective analysis. However, it is important to point out that, engage in PA once a week was still inversely associated with the depression score, supporting the idea that even little PA is beneficial. Furthermore, practicing PA at least once a week consistently over the years or only in the present is associated with a reduced risk of depressive symptoms when compared to those who practice less than once a week over the years.

Previous studies have found contradicting results considering sexes. For example, Mikkelsen, et al.^25^ reported that whereas women seem to be significantly at higher risk of depression when practicing low levels of PA, men did not. Contrary, other studies disclosed that men that did not achieve the recommended levels of PA had a higher likelihood of depression, but for women these results were less clear^26^. In this study, both men and women presented inverse associations between PA and the depression score, however, it is interesting to notice that the association was of a higher magnitude among women, especially in the prospective analysis. Notwithstanding, these study results demonstrate that PA is beneficial and has a similar impact on depression score reduction for both men and women.

Some limitations must be acknowledged. PA was self-reported, which is more likely to be biased than using an objective measurement^27^. Moreover, PA data indicate intensity and frequency, however, there is no information about the duration. This would be of importance to understand the effect PA duration in each session on depressive symptoms. Due to some factors, such as social desirability, there may be an intensity and frequency overestimation when respondents are questioned about their PA levels^28^. Furthermore, the self-report PA does not allow a clear distinction to be made between moderate PA and vigorous PA, because moderate PA and vigorous PA are mixed or not-separated. Additionally, the depression score measurement, although being a properly validated methodology^14^, might be susceptible to bias. For instance, mood fluctuations might influence participants’ responses. Another limitation is the lack of information regarding light intensity PA. There is some research considering that depression may limit late middle aged to older adults to be more physically active, and thus, light PA plays an important role, presenting a beneficial association with depression^29^.

Despite these limitations, the study presents some strengths. One of the major strengths is the use of a large sample of late middle aged to older adults from several European countries, which helps to mitigate the limitations of the indirect measurement of PA and depression. Furthermore, using a large European sample of late middle aged to older adults is considered relevant to provide strong evidence to policymakers, in order to strengthen the need for reflection upon the possible positive impact on society^30^. Lastly, the use of a cross-sectional and prospective design is recommended as it allows to better determine prevalence and to identify the causal associations between the studied variables^31,32^.

Considering the results of this study and its limitations, for future research, it would be interesting to further explore the role of PA intensity and frequency on the association with depression in late middle aged to older adults. It would be also interesting to test our findings with less bias and more objective measurements of PA and depression, using accelerometers and medical diagnosed depression. Future studies with large sample sizes should seek to extend the follow-up period with regular inter-assessment intervals to have a better understanding of time effects on the associating between PA and depression (Supplemenrat information S1).

## Conclusion

PA is inversely associated with depression, both cross-sectional and prospectively. From prospective analysis, it is clear that PA predict depression scores 4 years later. PA seems to provide positive effects on both prevention and treatment of depression. It is important to notice that it is enough to practice moderate PA to have a depression score reduction and that these results are significant for both men and women.

## Supplementary information

Supplementary file 1
