# Supplementary material for: Cross-sectional and prospective relationship between physical activity and depression symptoms
Source: Sci Rep. 2020 Sep 30;10:16114. doi: 10.1038/s41598-020-72987-4 (PMC7527477; doi:10.1038/s41598-020-72987-4)
Supplement: Supplementary file 1 [file 41598_2020_72987_MOESM1_ESM.docx]

**Suplementar material**

Supplementary table. Raw depression according to frequency and intensity of physical activity by sex

| 2011 | Men  Mean (95% CI) | *p* |  | Women  Mean (95% CI) | *p* |
| --- | --- | --- | --- | --- | --- |
| MPA  Less than once a week  Once a week  More than once a week | 2.63 (2.53, 2.73)  2.05 (1.96, 2.14)  1.78 (1.74, 1.81) | <0.001 |  | 3.75 (3.66, 3.84)  2.96 (2.87, 3.05)  2.60 (2.56, 2.64) | <0.001 |
| VPA  Less than once a week  Once a week  More than once a week | 2.23 (2.18, 2.29)  1.74 (1.65, 1.82)  1.67 (1.62, 1.71) | <0.001 |  | 3.17 (3.12, 3.22)  2.46 (2.39, 2.54)  2.43 (2.37, 2.48) | <0.001 |
|  |  |  |  |  |  |
| 2015 |  |  |  |  |  |
| MPA  Less than once a week  Once a week  More than once a week | 2.69 (2.59, 2.80)  2.05 (1.96, 2.14)  1.86 (1.82, 1.90) | <0.001 |  | 3.84 (3.75, 3.93)  3.03 (2.94, 3.12)  2.61 (2.57, 2.65) | <0.001 |
| VPA  Less than once a week  Once a week  More than once a week | 2.40 (2.37, 2.44)  1.62 (1.56, 1.67)  1.57 (1.53, 1.60) | <0.001 |  | 3.20 (3.17, 3.24)  2.41 (2.36, 2.47)  2.29 (2.25, 2.33) | <0.001 |
| Abbreviation: MPA, moderate physical activity; VPA, vigorous physical activity; CI, confidence interval | | | | | |
